# Zuogui Wan rescues the high-glucose-induced damaging effects on early embryo development

**DOI:** 10.1186/s12906-016-1151-8

**Published:** 2016-06-02

**Authors:** Temaka Bai, Qianjin Feng, Shien Zhu, Xin Niu, Yingli Wang, Kaixia Xu

**Affiliations:** School of Basic Medical Sciences, Beijing University of Chinese Medicine, Beijing, 100029 People’s Republic of China; School of Basic Medical Sciences, Shanxi University of Traditional Chinese Medicine, Taiyuan, 030024 People’s Republic of China; National Engineering Laboratory for Animal Breeding, Key Laboratory of Animal Genetics, Breeding and Reproduction of the Ministry of Agriculture, College of Animal Science and Technology, China Agricultural University, Beijing, 100193 People’s Republic of China

**Keywords:** Early embryo development, Blastocysts, Zuogui Wan

## Abstract

**Background:**

High concentration of glucose in culture medium affects the developmental process and the quality of the pre-implantation embryo. This study examined the effects of Zuogui Wan (ZGW) supplementation on early embryo development cultured in high-glucose medium.

**Methods:**

Embryos were cultured in high-glucose medium with or without ZGW supplementation. Developmental rate and competence was evaluated by cleavage rate, blastocyst rate, and blastocyst total cell number, reactive oxygen species (ROS) level, glutathione (GSH) concentration, and metabolome were also measured to determine the effect of ZGW on embryo development at the cellular level.

**Results:**

Compared with the vehicle group, supplementation of 0.01 % (v/v) ZGW to high-glucose medium significantly increased cleavage rate (80.1 ± 1.0 % vs 72.1 ± 1.3 %), blastocyst rate (50.5 ± 1.0 % vs 41.3 ± 1.7 %), and blastocyst total cell number (63.2 ± 2.2 vs 57.2 ± 1.6). ROS level was lower and GSH concentration in blastocysts was higher in ZGW-treated group. Metabolomic analysis found that the ratio of glucose to succinic acid and glucose to fumaric acid were lower in the ZGW-treated group .

**Conclusions:**

Developmental rates of zygotes in high-glucose culture medium were significantly lower than those in regular culture medium. ZGW supplementation significantly improved embryo development and quality in high-glucose medium. Supplementing ZGW in high-glucose medium also significantly increased total cell number and GSH concentration but decreased ROS level in blastocysts likely by modifying metabolic profile during embryo development. Together, these data suggest that supplementation of ZGW rescues high-glucose-induced detrimental effects on pre-implantation embryo development.

## Background

Pre-implantation embryo development can be affected by various factors including maternal diabetes. Maternal diabetes increases the risk of adverse pregnant outcomes, such as spontaneous abortion, intrauterine death and congenital malformations [1–4]. High-glucose level in the maternal environment induces early embryo loss, growth retardation and increases the percentage of congenital malformations, such as cardiovascular anomalies and neural tube defects [5–7]. Approximately 40 % of perinatal deaths are due to congenital malformations [6]. In addition, the offspring of diabetic mothers has a higher tendency to develop subsequent obesity, sustained impaired glucose tolerance and diabetes during adult life [7–9]. These findings revealed that high-glucose level can affect embryo development, embryo quality and long-term outcome after birth.

High-glucose concentration exert its detrimental effect on embryo development and survival by inducing DNA damage. High level of glucose increases oxidative stress by producing large amount of ROS, which can inhibit embryo development [7, 10, 11]. High-glucose level triggers DNA damage response through oxidative stress, finally results in abnormal development of embryo [12]. Addition of antioxidants such as proanthocyanidins can alleviate oxidative stress and prevent high glucose-induced malformation, but has no hypoglycemic effect [13–15].

Based on these data, we hypothesized that reducing ROS level in pre-implantation could decrease oxidative stress, therefore efficiently minimize the damaging impacts of high-glucose on embryo development.

ZGW was first introduced in by Jingyue Zhang (1563–1640 A.D.), a famous doctor of traditional Chinese medicine from Ming dynasty. According to the traditional Chinese medicine theory, ZGW has the efficacy of strengthening the body by means of nourishing Yin, enhancing the kidney function, replenishing essence, and promoting marrow content. Therefore, in the traditional application, ZGW is widely used in the treatment of diseases caused by the lacking of Yin and essence, such as gynecological disease-menstrual disorder, orthopedic disease-osteoporosis and most recently diabetes.

Pharmacological studies revealed that ZGW exhibits a wide range of effects. For example, ZGW can regulate the glucose metabolism [16–19]. ZGW treatment induces cells proliferation and differentiation [20], and inhibits cell apoptosis [21, 22]. The cellular role of ZGW may be due to its function as an antioxidant. To examine whether ZGW can promote early embryo development in high-glucose medium by reducing ROS and modulating the metabolic profile, we included vehicle or ZGW with various concentration and measured developmental rates, ROS level, GSH concentration, and metabolome. Embryo cultured in regular medium was used as a control. Our data suggest that ZGW can rescue the damaging effects of high-glucose on pre-implantation embryo development.

## Methods

All chemicals used in this study were purchased from Sigma Chemical Company (St. Louis, MO, USA), unless otherwise indicated. The Institution Animal Care and Use Committee at the China Agricultural University (Beijing, China) approved the protocols used in this study.

### Extraction of serum containing ZGW

Materials of ZGW (Rehmannia: Yam: Medlar: Cornus: Dodder: Antlet glue: Tortoiseshell glue: Radix cyathulae 8: 4: 4: 4: 4: 4: 4: 3) were purchased from Beijing Tongrentang pharmacy, and then decocted and extracted. The concentration is 1 g · mL^−1^ of dried medicinal herbs.

The rats were gavage feeding with ZGW extract for 7 days, 20 g ZGW/kg body weight per day. After the last gavage, blood was drawn from the abdominal aorta, and then all the blood samples were centrifuged to get serum.

### Collection and in vitro culture of murine embryos

ICR female mice were superovulated with 5I.U. PMSG followed 48 h later by 5I.U. hCG and mated overnight with ICR males of proven fertility. The mice with plug were labeled as pregnant mice (0.5d).

Zygotes were collected into M2 medium, cumulus cells were removed by gently pippetting in M2 medium containing 0.3 mg/ml hyaluronidase. For each repeat, 30 zygotes were collected and transferred into a droplet of 50 μL culture medium. The medium consisted of KSOM supplemented with ZGW. To examine the dose response of ZGW treatment, 0.01 %, 0.1 %, 1 % (v/v) ZGW was added to the medium. The zygotes were incubated at 37 °C in 5 % CO_2_ in air with saturated humidity. Cleavage and blastocyst formation rates were counted on Day 2 and Day 5, respectively.

### Analysis of total cell numbers in blastocysts

Day-5 blastocysts were incubated in M2 medium containing 10 μg/mL Hoechst 33,342 for 15 min at 37 °C in the dark, and then washed three times in M2 medium. Subsequently, the blastocysts were mounted on microscope slides and examined under an epifluorescence microscope to determine the total numbers of cell nucleus.

### Measurement of ROS and GSH levels

10 blastocysts were pooled to determine the ROS and GSH levels on Day 5. To measure ROS level, embryos were incubated in M2 medium supplemented with 10 μmol/L 20,70-dichlorodihydrofluorescein diacetate (H_2_DCFDA) for 20 min at 37 °C in the dark, washed three times in M2 medium, and then mounted on microscope slides and examined under an epifluorescence microscope with a filter at 460 nm excitation. Fluorescence images were recorded as TIFF files using a cooled CCD camera (DP72, Olympus, Tokyo, Japan). The recorded fluorescence intensities were quantified by EZ-C1 Free Viewer software (Nikon, Tokyo, Japan). The level of GSH in each blastocyst was measured with 10 μmol/L 4-chloromethyl-6.8-difluoro-7-hydroxycoumarin (Cell-Tracker Blue) with a filter at 370 nm excitation. The experimental procedure was same as the ROS measurement described above [23].

### Measurement of metabolome

The samples were extracted, and their metabolites profiles were analyzed by GC-TOF/MS as described by Jonas [24]. Briefly, embryos were cultured in high-glucose medium with ZGW or vehicle for 5 days, and then the metabolites were determined in the medium by GC-TOF/MS. The gas flow rate through the column was 1 ml min^−1^, the column temperature was held at 70 °C for 1 min, then increased by 5 °C min^−1^ to 280 °C, and held for 10 min. The transfer line and the ion source temperatures were 250 °C and 220 °C, respectively. Ions were generated by a 70 eV electron beam, and 10 spectra s^−1^ were recorded in the mass range 50–800 Da. The acceleration voltage was turned on after a solvent delay of 330 s. The detector voltage was 1600 V.

### Statistical analysis

Multivariate statistical analysis (MVSA) was performed using SIMCA 10.0 software (Umetrics, Umea°, Sweden). The data matrix was constructed by GC-TOF/MS responses of each peak as variables with the sample names/IDs as observations in columns and each of the peaks in rows. Subsequently, a principal component analysis (PCA), a partial least-squares-discriminant analysis (PLS-DA), and an orthogonal single collection partial least squares discriminant analysis (OSC-PLS) were used to process the acquired data from the GC-TOF/MS analysis. All data were analyzed in SPSS 19.0 software (SPSS Inc, Chicago, IL, USA) using one-way ANOVA. Differences were considered to be significant at *P* < 0.05. The results were expressed as means ± standard error of the mean.

## Results

### Effects of ZGW on developmental competence of embryo

To determine the effects of ZGW on developmental competence of embryo, embryos were cultured in high-glucose medium supplemented with vehicle only (high-glucose group), 0.01 %, 0.1 %, 1 % (v/v) ZGW for 5 days. As shown in Table 1, embryos cultured in the presence of 0.01 % (v/v) ZGW exhibited higher cleavage rate (80.1 ± 1.0 % vs 72.1 ± 1.3 %), blastocyst formation rate (50.5 ± 1.0 % vs 41.3 ± 1.7 %), and total cell numbers in blastocysts (63.2 ± 2.2 vs 57.2 ± 1.6) compared to those of vehicle. However, compared to the embryos cultured in regular medium, ZGW supplementation can’t completely rescue the detrimental effect of high-glucose on embryo development.

**Table 1:** Effects of ZGW on early embryo development in high-glucose medium

ZGW (%)(v/v)	Zygote (n)	Cleaved (%)	Blastocyst (%)	Cell numbers (n)
Regular medium	93	83.9 ± 1.5a	55.9 ± 1.0a	68.4 ± 2.4a
HG medium	97	72.1 ± 1.3be	41.3 ± 1.7b	57.2 ± 1.6be
HG medium (vehicle)	92	71.0 ± 1.6bc	38.0 ± 1.2c	54.0 ± 2.4c
HG medium(0.01 %)	95	80.1 ± 1.0d	50.5 ± 1.0d	63.2 ± 2.2d
HG medium(0.10 %)	94	73.4 ± 1.7e	44.8 ± 2.2e	60.0 ± 1.9e
HG medium(1.00 %)	92	69.6 ± 1.7c	39.1 ± 1.7bc	55.6 ± 2.6bc

Regular medium: KSOM medium with 0.2 mmol/L glucose

HG medium: high glucose medium (KSOM medium with 15.6 mmol/L glucose)

Vehicle: 0.01 % normal serum

Data are presented as mean ± S.E.M

Values with different superscript letters (a,b,c,d,e) within the same column are statistically significant (*P* < 0.05)

### Effects of ZGW on levels of ROS and GSH levels in embryos

**Fig. 1:**
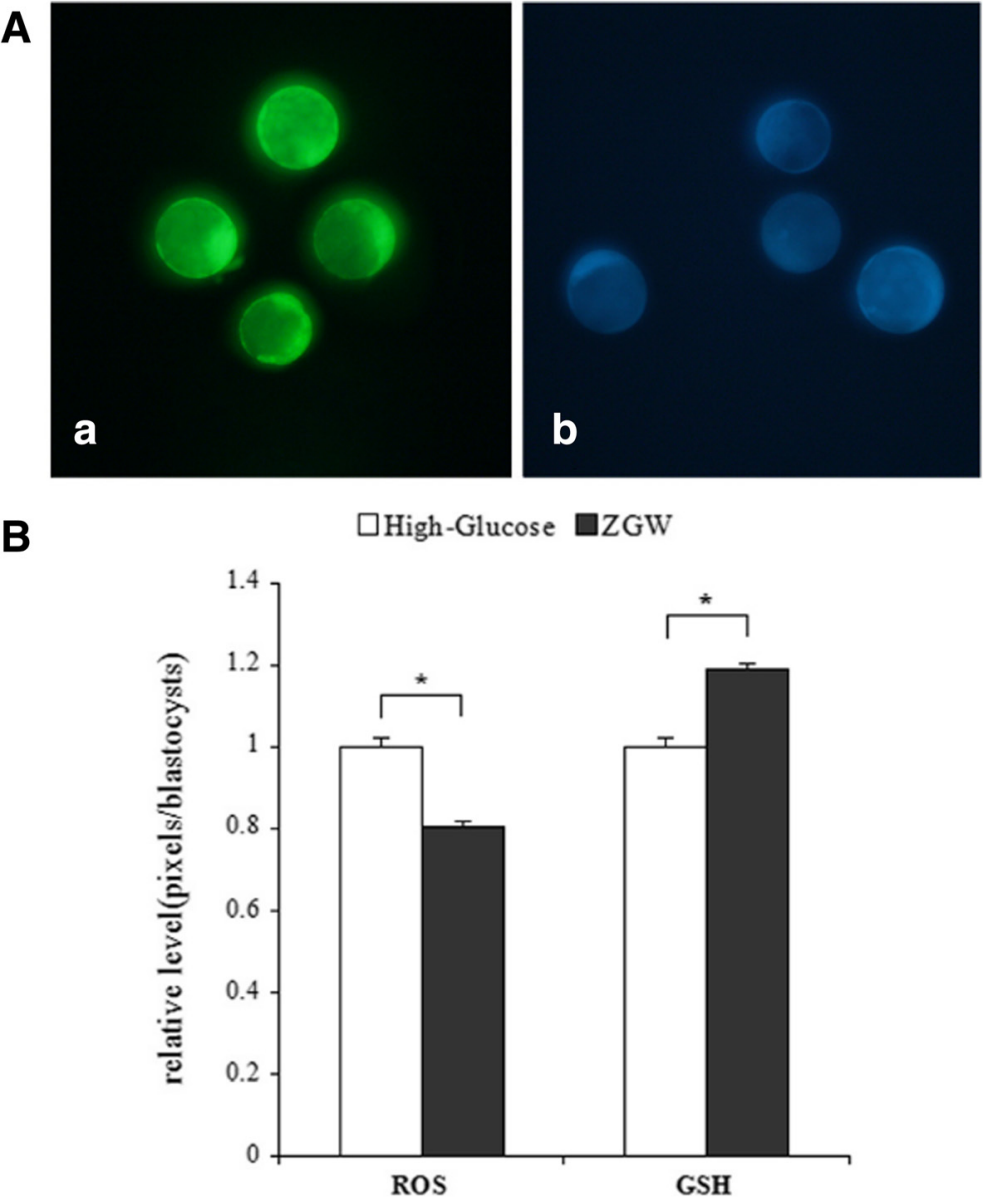
ROS and GSH levels in blastocysts treated with ZGW or vehicle. **a** Representative fluorescence photomicrographs of blastocysts stained with H2DCFDA (**a**) and CellTracker Blue (**b**); **b** Quantification of ROS and GSH levels by fluorescence intensities. Data are presented as mean ± S.E.M. Asterisks indicate a significant difference between the ZGW-treated group and the high-glucose group (*P* < 0.05)

Embryos were cultured in high-glucose medium with or without ZGW for 5 days, and then the levels of ROS and GSH were determined in the blastocysts. As shown in Fig. 1, addition of 0.01 % (v/v) ZGW to high-glucose medium decreased ROS level by 19.4 % in blastocysts while increased GSH level by 19.1 % with the vehicle.

### Effects of ZGW on metabolome

**Fig. 2:**
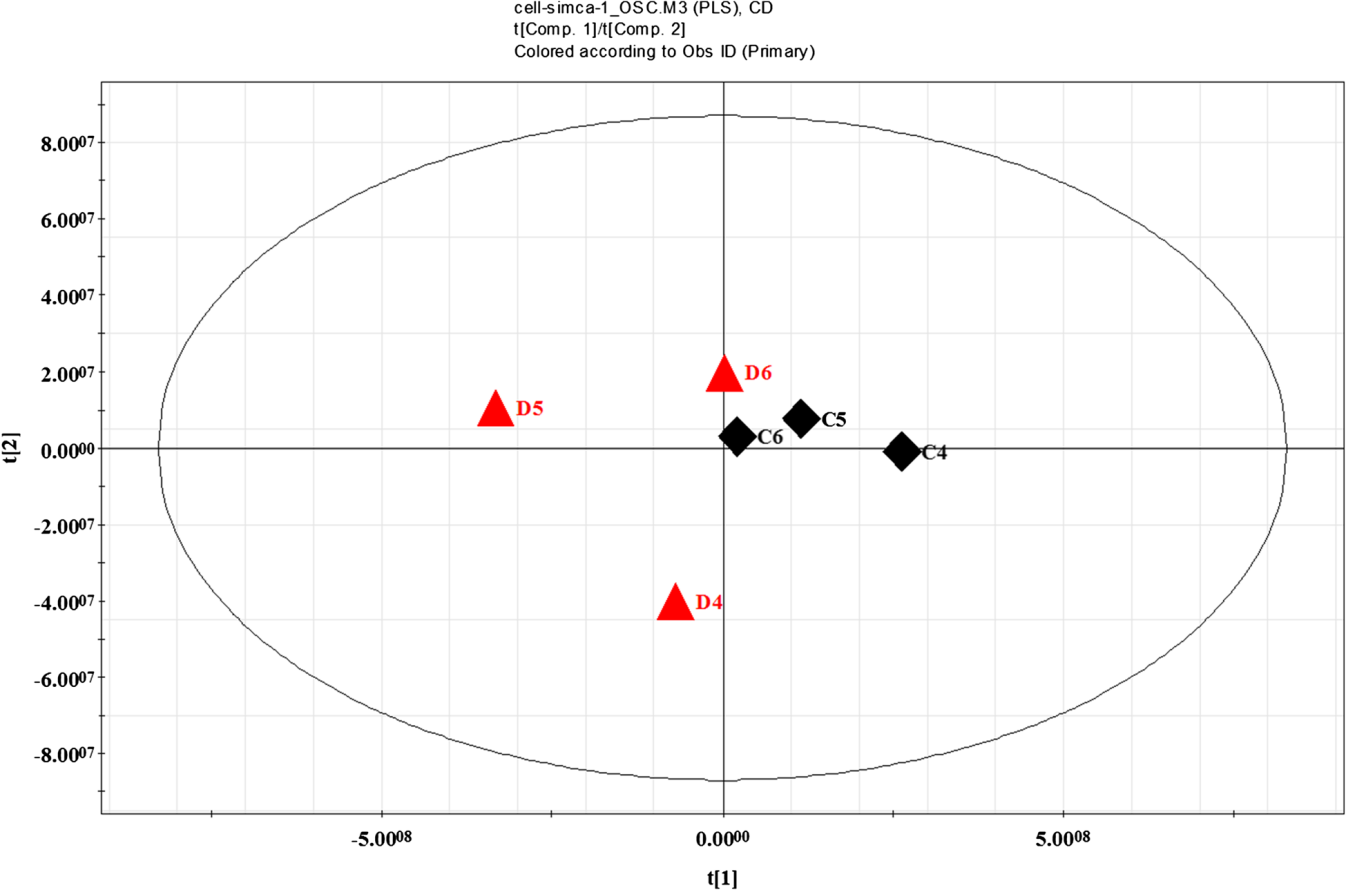
ZGW-treated high-glucose vs high-glucose. OSC-PLS scatter scores. high-glucose medium supplemented with ZGW: C4,C5,C6; high-glucose medium: D4,D5,D6

**Fig. 3:**
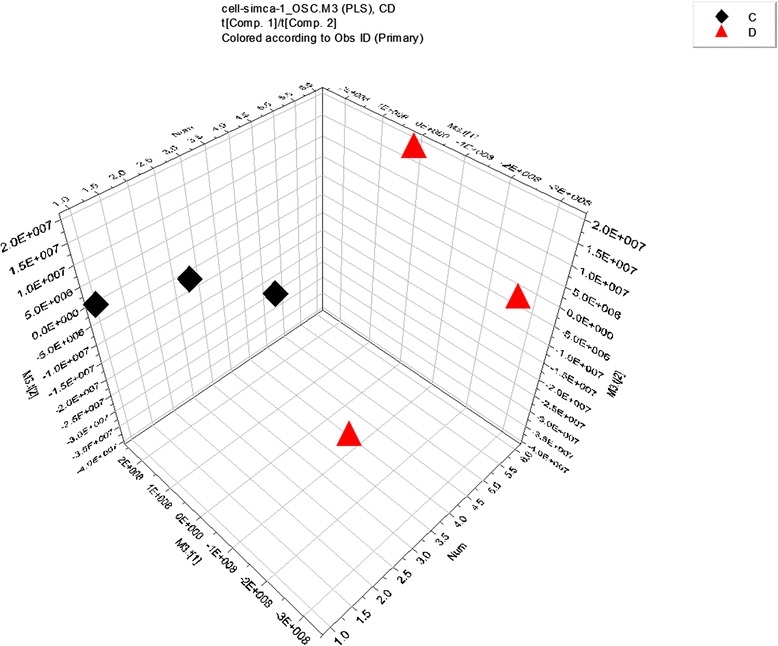
ZGW-treated high-glucose vs high-glucose. OSC-PLS 3D scatter scores. high-glucose medium supplemented with ZGW: C4,C5,C6;high-glucose medium: D4,D5,D6

**Fig. 4:**
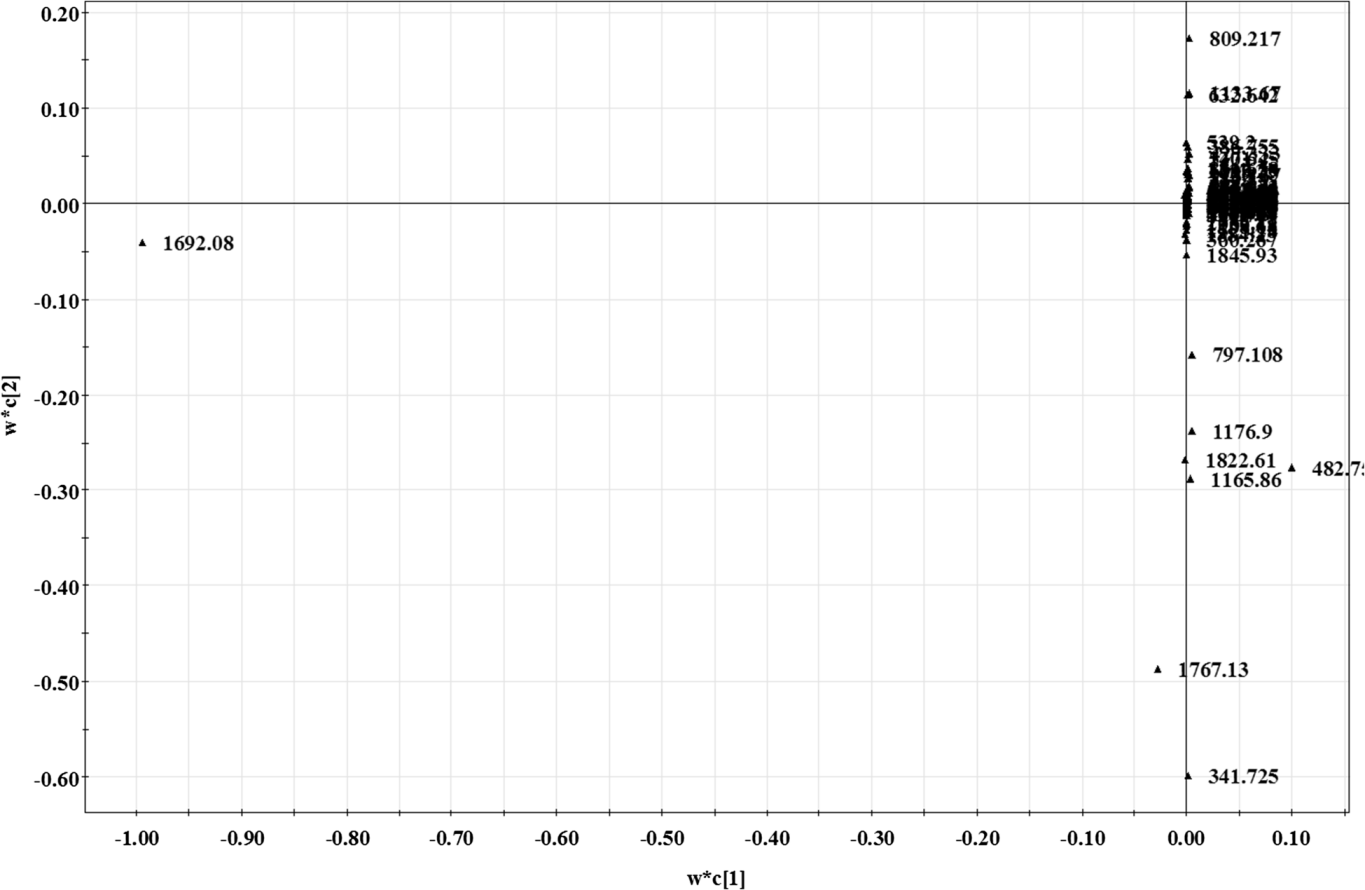
ZGW-treated high-glucose vs high-glucose. OSC-PLS load diagram

By using GC TOF/MS, we detected a total of 352 putative metabolites, of which 49 were identified. As shown in Figs. 2, 3 and 4, The samples from ZGW-treated group show a clear trend of separation compared to the samples from vehicle group, indicating the metabolites between two groups were strikingly different. ZGW supplementation modified metabolic profile during embryo development. We calculate the content of glucose, succinic acid and fumaric acid, the ratio of glucose to succinic acid and glucose to fumaric acid. We find addition of 0.01 % (v/v) ZGW to high-glucose medium significantly decreased the ratio of glucose to succinic acid and glucose to fumaric acid in medium compared with the high-glucose group.

## Discussion

Glucose provides energy to organism by metabolic product ATP, participates in energy metabolism and supports growth and development. Interestingly, embryos from maternal diabetes or cultured in high-glucose medium show abnormal development [12]. High-glucose level results in retardation of embryo development, congenital malformations, and even spontaneous abortion [5–7]. It was revealed that high-glucose level increases oxidative stress by producing large amount of ROS, therefore inhibit embryo development [7, 10, 11]. Previous studies prove that addition of antioxidants such as vitamin C, vitamin E and proanthocyanidins can reduce the detrimental effects of high-glucose, but cannot effectively decrease high-glucose level [7, 15].

Our results revealed that, under the high-glucose stimulation, supplementation of 0.01 % (v/v) ZGW significantly improved the proportion of embryo development in terms of cleavage rate, blastocyst formation rate, and total cell numbers in blastocysts. Although 0.1 %, 1 % (v/v) ZGW was not fully effective, the optimal dosage of ZGW improves embryo quality morphologically, this may due to its role as a strong antioxidant. Previous studies found that ZGW can improve the activities of superoxide dismutase (SOD), catalase (CAT) and glutathione peroxidase (GSH-PX), decrease the lipid peroxide (LPO) level, thereby give arise of antioxidation [25]. Reducing elevated oxidation in high-glucose condition may increase cell membrane integrity, and indirectly reduce cells apoptosis [21, 22], therefore promoting early embryo development. However, ZGW can’t fully rescue embryo development in high-glucose medium, indicating high-glucose affects embryo development in a multifunctional way.

ZGW also modulates the metabolic profile of mouse blastocysts. Our results revealed that, supplementation of 0.01 % (v/v) ZGW had a significant decrease in ratio of glucose to succinic acid and glucose to fumaric acid compared with the high-glucose group. Tricarboxylic acid cycle (TCA cycle) is the main route of energy metabolism for organism. Glucose as starting material, pyruvic acid, succinic acid and fumaric acid are important intermediates in TCA cycle. Therefore the overall metabolism outcome of TCA cycle is that glucose level decreases, and levels of succinic acid and fumaric acid increase simultaneously. The smaller the ratio of glucose to succinic acid or fumaric acid is, the higher the use ratio of glucose is. This analytical method may provide a better evaluation of dynamic changing of glucose during metabolism.

In summary, ZGW can promote aerobic metabolism route of glucose, thereby significantly improve glucose use capacity of embryo, and increase glucose consumption rate. Therefore the glucose concentration is significantly reduced, which decreases the high-glucose level. Therefore ZGW can efficiently decrease oxidative stress and improve the proportion of embryo development and quality of embryo.

## Conclusion

We demonstrated that, under the high-glucose stimulation, supplementation of 0.01 % (v/v) ZGW enhances the developmental competence of embryo. Developmental rates of zygotes in high-glucose culture medium were significantly lower than those in regular culture medium. Embryos cultured in high-glucose medium supplemented with ZGW showed similar developmental rates to those in regular medium. Supplementing ZGW in high-glucose medium also significantly increased total cell number and GSH concentration but decreased ROS level in blastocysts. Together, these data suggest that supplementation of ZGW rescues high-glucose-induced detrimental effects on pre-implantation embryo development.

## Abbreviations

CAT, catalase; GSH, glutathione; GSH-PX, glutathione peroxidase; hCG, human chorionic gonadotropin; LPO, lipid peroxide; PMSG, pregnant mare serum gonadotropin; ROS, reactive oxygen species; SOD, superoxide dismutase; TCA cycle, tricarboxylic acid cycle; ZGW, Zuogui Wan
